# Interaction of *Serratia proteamaculans* with Integrins Activates Invasion-Promoting Signaling Pathways

**DOI:** 10.3390/ijms26093955

**Published:** 2025-04-22

**Authors:** Olga Tsaplina

**Affiliations:** Institute of Cytology, Russian Academy of Sciences, Tikhoretsky av. 4, 194064 St. Petersburg, Russia; tsaplina@incras.ru; Tel.: +7-(812)297-42-96

**Keywords:** RGD peptide, α5β1 integrin, EGFR, c-Src kinase, focal adhesion kinase (FAK), integrin-linked kinase (ILK)

## Abstract

The opportunistic bacteria *Serratia proteamaculans* are able to penetrate human cells. It was previously shown that the bacterial surface protein OmpX promotes bacterial adhesion. In addition, infection with bacteria that synthesize the OmpX protein enhances the expression of EGFR and β1 integrin involved in the invasion of *S. proteamaculans*. Therefore, this work was aimed at determining the mechanism of interaction of *S. proteamaculans* with receptors of eukaryotic cells. Both integrin-linked kinase (ILK) and EGFR tyrosine kinase have been shown to be involved in the invasion of these bacteria. During infection, EGFR is first phosphorylated at Tyr845, which is carried out by c-Src kinase transmitting a signal from nearby receptors. The *S. proteamaculans* invasion depends on c-Src and focal adhesion kinase (FAK), which can both transmit a signal between β1 integrin and EGFR and participate in cytoskeletal rearrangements. These bacteria have been shown to interact with integrin not through the RGD binding site, and integrin binding to the RGD peptide enhances adhesion, invasion, and expression of α5 and β1 integrin subunits in response to infection. On the other hand, bacterial adhesion and increased expression of integrins during infection are caused by OmpX. Thus, OmpX interacts with integrins, and the participation of the α5 and β1 integrin subunits in the *S. proteamaculans* invasion allows us to assume that the receptor of OmpX is α5β1 integrin.

## 1. Introduction

*Serratia* are opportunistic bacteria, causing infections in plants, insects, animals and humans under certain conditions [1]. *Serratia marcescens* and *Serratia liquefaciens* have been isolated as agents in numerous outbreaks and opportunistic infections associated with point sources such as medical devices and various solutions given to hospitalized patients. *Serratia proteamaculans* belongs to the *S. liquefaciens* complex [1]. To evade host immune cells, bacteria have developed an infection-promoting strategy involving invasion of non-phagocytic cells. We have previously shown that the *S. proteamaculans* can also invade eukaryotic cells [2]. Inactivation of the intracellular protease protealysin leads to an increase in the intensity of invasion of these bacteria [3]. This indicates the existence of virulence factors among substrates of protease protealysin. One of the identified protealysin substrates is the bacterial surface protein OmpX [3]. OmpX family proteins have shown the ability to bind to the epidermal growth factor receptor (EGFR) [4] and extracellular matrix protein fibronectin [5], which is an adapter for bacterial binding to the α5β1 integrin on the surface of eukaryotic cells [6]. We have shown that contact with the OmpX protein of *S. proteamaculans* causes an increase in the expression of EGFR and β1 integrin [7], and these receptors are involved in the invasion of *S. proteamaculans* [7]. However, it is unknown whether the interaction with these receptors on the surface of the host cell occurs independently or whether signal transmission in the infected cell involves binding to one receptor and transmitting the signal through a nearby receptor. Therefore, this work was aimed at determining the mechanism of interaction of *S. proteamaculans* with the receptors of eukaryotic cells.

Integrins mediate a variety of functions through ligand binding, receptor clustering, or both [8]. The short cytoplasmic domains of integrins have no endogenous kinase activity of their own; they must bind accessory molecules that contribute cytoskeletal reorganizing and signal transduction [9]. Integrin clustering recruits adapter and signaling proteins into multiprotein cytoplasmic adhesion complexes called focal adhesions, which activate signaling pathways that lead to changes in gene expression [10]. In addition, integrins can use the EGFR component of this complex as a transmitter in the signaling pathways downstream of the cell matrix [11]. In the case of integrin-dependent EGFR-phosphorylation, activation is dependent on the c-Src associated with the multimeric integrin–EGFR complex [12]. c-Src-mediated phosphorylation of EGFR Tyr845 is involved in the regulation of receptor function [13]. The integrin activation induces clustering of EGFR [14]. The interaction between integrins and growth factor receptors is well known as a coordinated mechanism used by cells to organize responses to external stimuli [15]. The possibility that *S. proteamaculans*, by binding to β1 integrin, triggers EGFR signaling to promote invasion is suggested by the fact that disruption of the membrane rafts by MβCD reduces its invasion [16], because the interaction between the EGFR and β1 integrin is known to be disrupted by MβCD [17]. This mechanism of invasion is described in most detail for *Campylobacter jejuni*. *C. jejuni* binding to host-cell integrins via the CadF and FlpA fibronectin-binding proteins activates the epidermal growth factor (EGF) pathway [18]. However, fibronectin is not involved in the invasion of *S. proteamaculans* [7]. This is the only difference in invasion between *S. proteamaculans* and *C. jejuni* that has been identified to date. All other host-cell components involved in *S. proteamaculans* invasion are identical to those involved in *C. jejuni* invasion. This is surprising given that invasion mechanisms can vary significantly even among different strains of the same bacterial species.

In this work, α integrin was identified, which forms a heterodimer with β1 integrin that interacts with *S. proteamaculans*. Inhibitor studies have revealed that the *S. proteamaculans* host-cell invasion was dependent upon c-Src, focal adhesion kinase (FAK), and integrin-linked kinase (ILK), all of which are known to participate in cytoskeletal rearrangements. The EGFR has been shown to be first phosphorylated at Tyr845. Phosphorylation of the EGFR at Tyr845 is independent of substrate binding and is mediated by c-Src [19,20]. This indicates that the EGFR mediates invasion by transducing a signal from a nearby receptor. Using the RGD peptide, it was shown that on the surface of the host cell, the *S. proteamaculans* does not interact with integrin through the RGD binding site, and the binding of the integrin to the RGD increases the response of the host cell to infection.

## 2. Results

### 2.1. EGFR Signaling During S. proteamaculans Invasion

**Figure 1:**
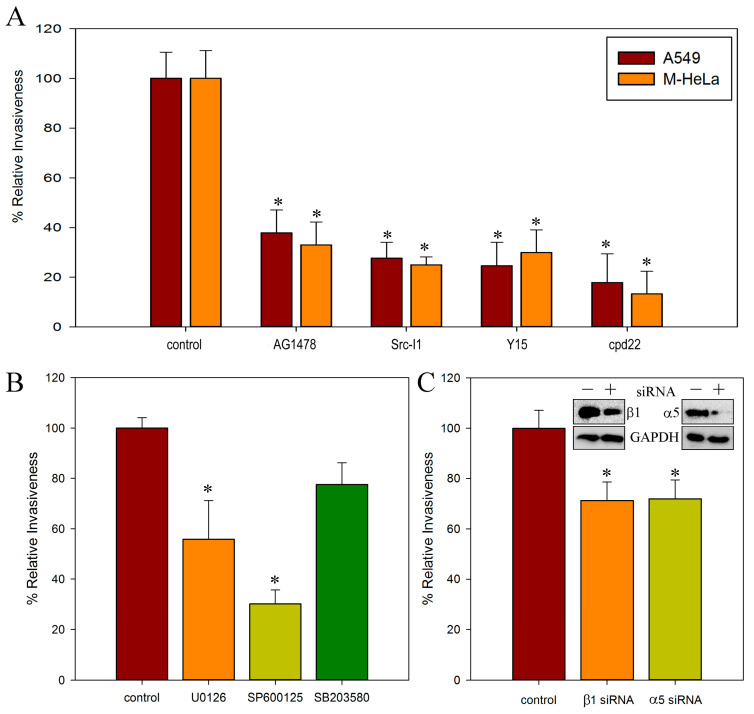
Involvement of integrins in *S. proteamaculans* invasion. (**A**) Comparison of the effect of inhibitors on the sensitivity of A549 and M-HeLa cells to bacteria. The sensitivity to bacteria of cells pre-incubated with inhibitor of EGFR (30 μM AG1478), c-Src kinase (5 μM Src I1), FAK (5 μM Y15) for 1 h, and ILK (2.5 μM cpd22) for 24 h was assessed quantitatively. Control—intensity of invasion into untreated cells. (**B**) Comparison of the effect of MAP kinase signaling pathway inhibitors on the sensitivity of M-HeLa cells to bacteria. The sensitivity to bacteria of cells pre-incubated with inhibitor of Raf/MEK/ERK (10 μM U0126), JNK (10 μM SP600125), p38 (5 μM SB203580) for 1 h was assessed quantitatively. Control—intensity of invasion into untreated cells. (**C**) Comparison of the effect of treating M-HeLa cells with siRNA targeting β1 and α5 integrins on cell sensitivity to *S. proteamaculans*. Pretreating cells with an siRNA reduced the β1 and α5 integrins expression by 46% and 25%, respectively. Control—M-HeLa cells transfected with siRNA containing scrambled nucleotide sequence. The insert shows the total amount of integrins and internal control GAPDH in untreated M-HeLa cells and pretreating cells with small interfering RNA targeting β1 and α5 integrins. The number of intracellular bacteria was estimated as a percentage, taking the number of intracellular bacteria in control samples as 100%. Values are expressed as mean S.D. (error bars) of a representative experiment. The difference in the control was considered significant at the * *p* < 0.05 level.

**Figure 2:**
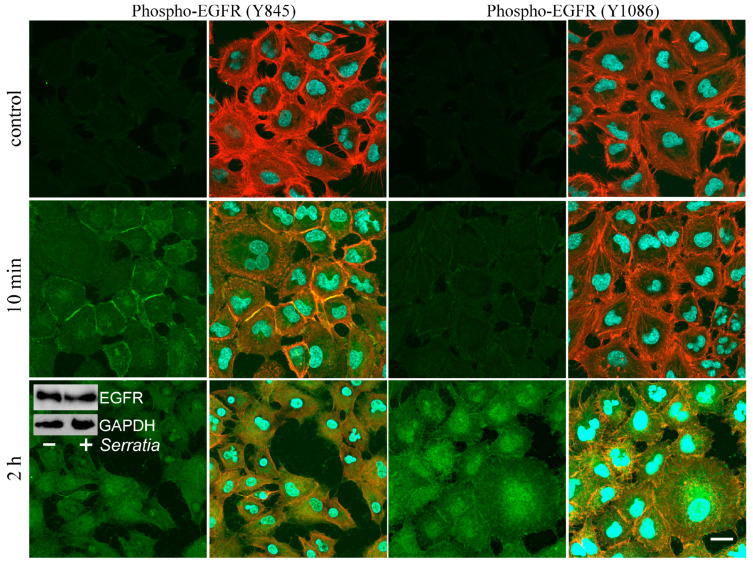
Distribution of phospho-EGFR in A549 cells as a result of incubation with *S. proteamaculans*. Cells were incubated with bacteria for 10 min or 2 h after bacteria sedimentation by centrifugation. Control—uninfected A549 cells. Cytoskeleton was stained with rhodamine-phalloidin (red); Phospho-EGFR was stained with antibodies (green); DNA was stained with DAPI (blue). Scale bar: 30 μm. The insert shows a total amount of EGFR and internal control GAPDH in A549 cells incubated for 2 h in absence (“−”) or presence (“+”) of *S. proteamaculans*.

Previously, based on the receptors’ redistribution, we made the assumption that different receptors can play a decisive role in the invasion of *S. proteamaculans* depending on the origin of the cell line [21]. It was also assumed that the intensity of invasion in A549 cells is determined by the EGFR, and in M-HeLa, cells it is determined by β1 integrin [21]. Therefore, we compared the contribution of EGFR to A549 and M-HeLa cell invasion. Using invasion quantification, we showed that an EGFR inhibitor reduced by 3-fold the sensitivity of both cell lines to bacteria (Figure 1A). Based on these data, we propose that the role of the EGFR in M-HeLa and A549 cells is similar. It has been previously shown that *S. proteamaculans* infection leads to the accumulation of EGFR in M-HeLa cells [7]. However, according to Western blot analysis in A549 cells, the total amount of EGFR does not change during infection (Figure 2, insert). To transmit a signal from the EGFR, its transition to a phosphorylated form is necessary. The cytoplasmic domain of the EGFR is rich in phosphorylation sites specific for various kinases. It has been shown that during the process of invasion, activation of the receptor can occur both as a result of phosphorylation of the Tyr845 residue by c-Src kinase [19,20], and by autophosphorylation of tyrosine at the Tyr1086 or Tyr1173 residues [19,20]. The redistribution of phospho-EGFR was assessed in A549 cells, in which EGFR redistribution is more obvious. Using confocal microscopy, we showed that after 10 min of incubation with bacteria, only EGFR phosphorylated at Tyr845 accumulates in A549 cells (Figure 2). And after 2 h of incubation with bacteria, EGFR is detected in the cells, phosphorylated at both Tyr845 and Tyr1086. Thus, tyrosine at Tyr845 residue, which is the target of c-Src kinase, is phosphorylated first. Subsequently, the tyrosine residue at Tyr1086 is phosphorylated as a result of autophosphorylation. Based on this, it can be assumed that the EGFR mediates *S. proteamaculans* invasion not by directly binding to bacterial surface proteins, but by transmitting a signal from other receptors of the infected cell.

The EGFR regulates numerous cellular activities such as apoptosis, gene expression, mitosis, differentiation, and immune responses via signaling through mitogen-activated protein kinases (MAP kinases). The MAP kinase family consists of major subfamilies of related proteins: extracellular-regulated kinases (ERK), c-Jun N-terminal kinase (JNK), and p38 [22]. The main data indicate that phosphorylation of the EGFR at Tyr845 triggers the ERK signaling pathway [23], but activation of the p38 [24] and JNK signaling pathways [25] has also been shown. Therefore, using inhibitory analysis, we quantitatively compared the contributions of these signaling pathways to the regulation of *S. proteamaculans* invasion (Figure 1B). We showed that inhibition of the ERK and JNK signaling pathways reduces invasion by 40% and 70%, respectively, and the p38 inhibitor has minimal effect on invasion intensity.

### 2.2. Involvement of c-Src, FAK, and ILK Kinases in S. proteamaculans Invasion

Phosphorylation of EGFR at Tyr845 is ligand independent and requires activation of c-Src. We therefore assessed the contribution of c-Src to *S. proteamaculans* invasion. To determine the contribution of c-Src kinase to bacterial invasion, we assessed the effect of a kinase inhibitor on the sensitivity of M-HeLa and A549 cells to bacteria (Figure 1A). Indeed, the c-Src kinase inhibitor reduces the intensity of invasion into the cells of both cell lines by 4 times. c-Src kinase is involved in signal transmission through EGFR from integrins [26] during bacterial invasion [19].

In carcinoma cells, integrin can trigger signal transmission from the EGFR through both ligand-dependent and ligand-independent pathways. It was assumed that signal transmission from integrins through the EGFR should be blocked by an inhibitor of c-Src kinase in a ligand-independent pathway and by an inhibitor of FAK kinase in a ligand-dependent pathway [26]. However, inhibitor analysis showed that the inhibitor of FAK, as well as the inhibitor of c-Src, reduced the intensity of invasion of *S. proteamaculans* into cells of both cell lines by 4 times (Figure 1A). On the other hand, the signal from integrin can be transmitted by ILK. We showed that the ILK inhibitor reduced the intensity of *S. proteamaculans* invasion in both cell lines by 6–8 times (Figure 1A). Thus, both EGFR and ILK signaling are required for *S. proteamaculans* invasion.

### 2.3. Role of α5β1 Intergin and Its RGD Binding Site in Invasion

We previously showed that β1 integrin is involved in *S. proteamaculans* invasion. Integrin is formed by a heterodimer of subunits α and β. Depending on the α integrin subunit with which the β1 integrin heterodimer is formed, it can bind to various substrates. By quantifying the invasion, we showed that siRNA against the α5 and β1 integrin subunits similarly reduced the sensitivity of M-HeLa cells to *S. proteamaculans* invasion (Figure 1C). Thus, α5β1 integrin is involved in the invasion of *S. proteamaculans*.

**Figure 3:**
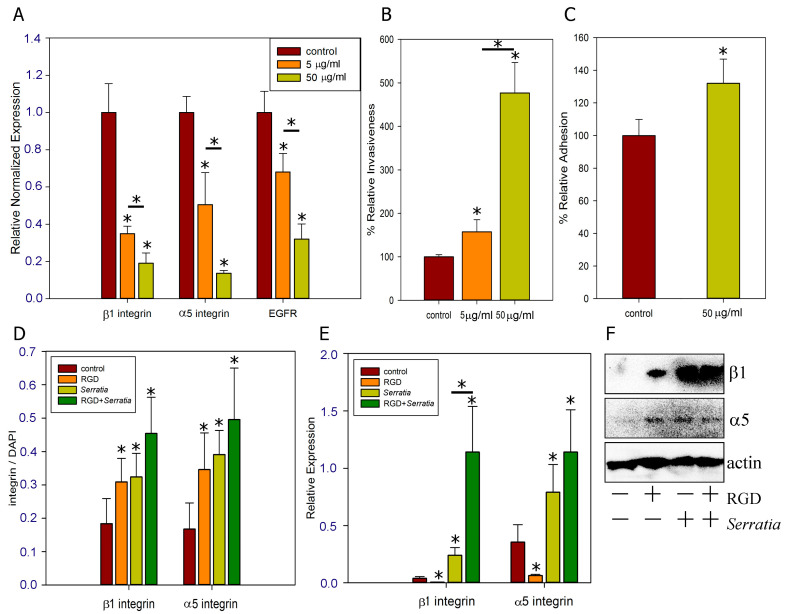
Effect of RGD peptide on *S. proteamaculans* invasion. (**A**) Effect of incubation with RGD peptide (5 μg/mL and 50 μg/mL) on receptor expression in M-HeLa cells. (**B**) Effect of pre-incubation of M-HeLa cells with RGD peptide (5 μg/mL and 50 μg/mL) for 30 min on the intensity of bacterial invasion. (**C**) Effect of pre-incubation of M-HeLa cells with 50 μg/mL RGD peptide for 30 min on the intensity of bacterial adhesion. (**D**) Effect of bacterial infection on amount of α5 and β1 integrin in the host cell. The mean fluorescence intensity of integrin normalized to the mean fluorescence intensity of DAPI estimated with ImageJ2 Fiji on 6–9 images obtained using confocal microscopy (Figure 4 shows an example). Values are expressed as mean S.D. (error bars). The difference to the control was considered significant at the * *p* < 0.05. (**E**,**F**) Effect of incubation with 50 μg/mL RGD peptide, *S. proteamaculans*, or co-incubation with RGD peptide and bacteria on expression (**E**) and total amount (**F**) of α5 and β1 integrins in M-HeLa cells.

α5β1 integrin binds to the RGD motif of fibronectin [27]. Therefore, the RGD peptide is widely used to show that bacteria bind specifically to the α5β1 integrin [28,29]. The RGD peptide inhibits invasion of host cells by *Bordetella pertussis* via integrin α5β1 [28] and by Group B *Streptococci* if they synthesize the fibronectin-binding protein sfbA [29]. We have shown that in addition, the binding of RGD peptide reduces the expression of α5β1 integrins and EGFR involved in *S. proteamaculans* invasion (Figure 3A). However, the decrease in expression cannot lead to a decrease in the amount of protein in 2.5 h of incubation. Conversely, the intensity of *S. proteamaculans* invasion into M-HeLa cells increased 5-fold in the presence of the RGD peptide (Figure 3B). At the same time, this peptide increases the intensity of bacterial adhesion by 30% (Figure 3C). On the one hand, it indicates that the increase in adhesion occurs as a result of the increase in the affinity of the bacterial protein with integrins in the presence of the RGD peptide. These data indicate that RGD-containing proteins (extracellular matrix proteins) interacting with integrins can enhance *S. proteamaculans* invasion if they do not shield the bacterial binding site, as shown for fibronectin [7]. On the other hand, an increase in adhesion by 30% is not enough to explain a 5-fold increase in invasion.

**Figure 4:**
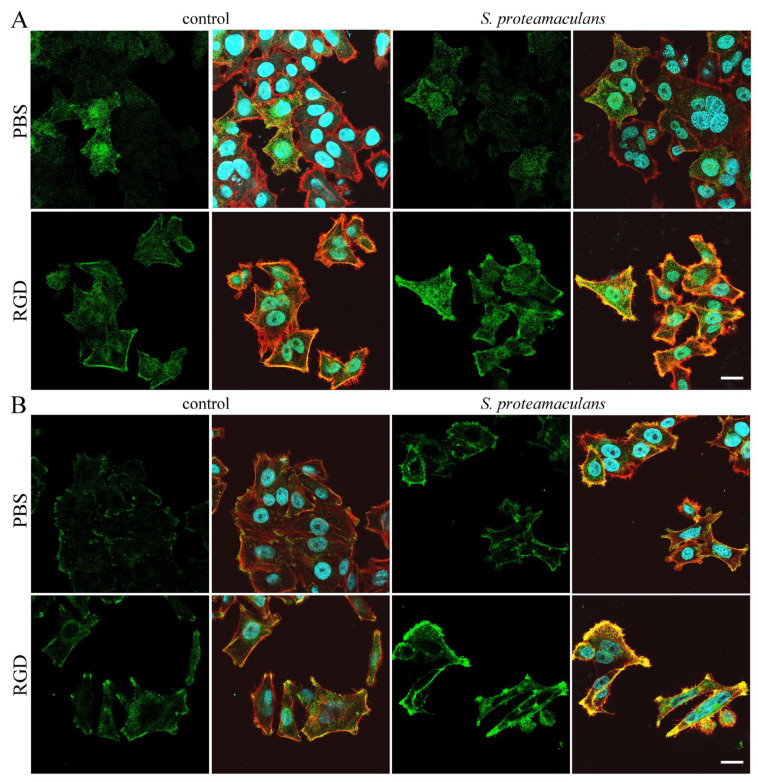
Distribution of α5 (**A**) and β1 (**B**) integrins in M-HeLa cells as a result of incubation with 50 μg/mL RGD peptide, *S. proteamaculans*, or co-incubation with RGD peptide and bacteria. A total of 50 μg/mL RGD peptide was added to the cells for 30 min, then the bacteria were pelleted by centrifugation onto the cell surface and incubated for 2 h. Control—uninfected M-HeLa cells. Cytoskeleton was stained with rhodamine-phalloidin (red); receptors were stained with antibodies (green); DNA was stained with DAPI (blue). Scale bar: 30 μm.

We hypothesized that the RGD peptide may cause receptor redistribution in the host cell. Using confocal microscopy, we showed that co-incubation with the RGD peptide and bacteria leads to the accumulation of integrins around the cell perimeter (Figure 4). Moreover, analysis of confocal microscopy data (Figure 3D) and Western blot analysis (Figure 3F) showed that treatment of cells with the RGD peptide leads to the accumulation of α5 and β1 integrin subunits. These integrin subunits are involved in invasion and their accumulation may lead to increased bacterial penetration into host cells. Using RT-PCR, we showed that preincubation of cells with RGD peptide leads to an even greater increase in integrin expression in response to infection with *S. proteamaculans* (Figure 3E). We suggest that the additional enhancement of receptor expression upon addition of RGD with bacteria may provide enhanced invasion. Thus, on the surface of the host cell, the bacteria *S. proteamaculans* does not interact with α5β1 integrin through the RGD binding site. Apparently, the RGD peptide changes the structure of the integrin such that after binding to RGD peptide, the integrin interacts more closely with bacteria. Thus, the RGD peptide increases the response of the host cell to infection.

### 2.4. Effect of Different Multiplicities of Infection (MOI)

**Figure 5:**
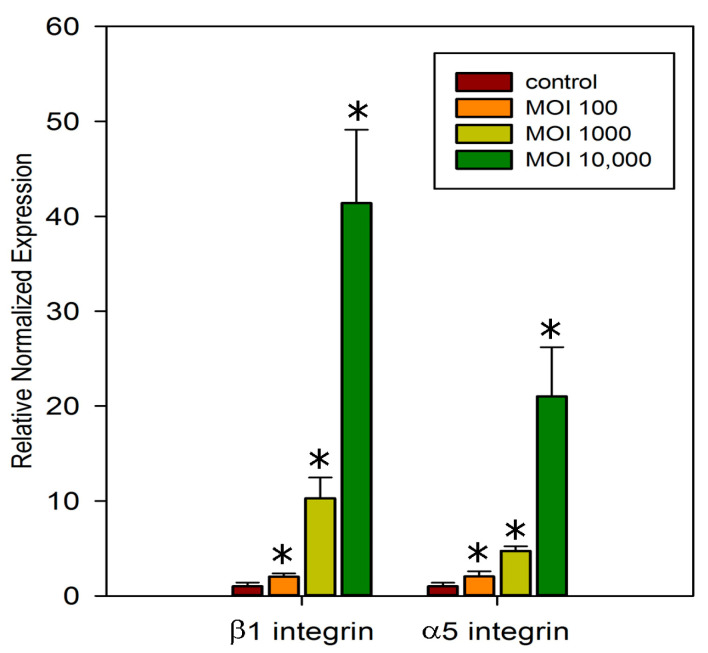
Comparison of infection with different multiplicities of infection (MOI) on integrins expression. Values are expressed as mean S.D. (error bars) of a representative experiment. The difference to the control was considered significant at the * *p* < 0.05 level.

Bacterial infection causes an increase in the expression of integrins α5 and β1 (Figure 3D). Integrin expression intensity increases with increasing multiplicity of bacterial infection (Figure 5). Moreover, the maximum intensity of *S. proteamaculans* invasion was observed when 1000 bacteria per cell were infected, and a 10-fold increase or decrease in the number of bacteria per cell reduced the invasion intensity by 4 times [30]. At an MOI of 10,000, bacteria infect 20–30% of cells in 2 h, and the total intensity of integrin expression increases up to 40 times. From this, we can conclude that the increase in integrin expression is due to the contact of the bacterial protein with the surface of the host cell, and not invasion. Moreover, as shown earlier, infection with *E. coli* does not affect the expression of integrins, and after transformation with a plasmid with the *ompX* gene of *S. proteamaculans*, the bacteria cause an increase in adhesion and receptor expression, although they do not become invasive [7]. And, based on the fact that pre-incubation with RGD peptide increases the number of adherent bacteria by 30% and increases the expression of α5β1 integrin several-fold during bacterial infection, we assume that the binding of the RGD peptide increases its affinity for OmpX with the host-cell integrins. Moreover, the addition of RGD results in changes in the conformation of α5β1 integrin, facilitating receptor binding to a ligand lacking the RGD domain. And the participation of α5 and β1 integrin subunits in the *S. proteamaculans* invasion allows us to assume that the receptor of OmpX is α5β1 integrin.

## 3. Discussion

### 3.1. EGFR in Bacterial Invasion

The EGFR, upon binding to the ligand, dimerizes and activates cytoplasmic tyrosine kinase, thereby stimulating signaling pathways that regulate cellular reactions [31]. In addition, EGFR is involved in the infection of host cells by bacterial pathogens through various mechanisms. Exogenous EGF can enhance the invasion of host epithelial cells by *Haemophilus influenzae*, thereby suggesting the involvement of EGFR signaling in invasion [32]. EGFR can either directly interact with bacterial surface proteins or play the role of an intermediary in signal transduction. The *Salmonella* Rck outer membrane protein directly binds to EGFR without competing with EGF [4]. And *Campylobacter jejuni* infection leads to the assembly of integrin-dependent signaling complexes and, as a result, phosphorylation and transactivation of EGFR [19]. *Helicobacter pylori* infection is associated with EGFR activation through the metalloproteinase-dependent release of EGFR ligands in gastric epithelial cells [33]. *Neisseria gonorrhoeae* also activates the EGFR without direct binding to it, stimulating gene expression and the surface cleavage of EGFR ligands [34]. One of the EGFR ligands cleaved from surface proteins is soluble E-cadherin (sE-cad). sE-cad can bind to EGFR and stimulate EGFR phosphorylation, playing a role in EGFR signaling independent of traditional EGFR ligands [35]. However, during the incubation of M-HeLa cells with *S. proteamaculans*, E-cadherin is cleaved. In response to S. *proteamaculans* infection, the EGFR is localized to bacteria-free endosomes [7]. Using siRNA targeting EGFR, its involvement in invasion was demonstrated [7]. Using inhibitors, we demonstrated the involvement of the EGFR tyrosine kinase domain and EGFR-triggered MAPK signaling cascades in the invasion. The main contribution to the *S. proteamaculans* invasion of these bacteria is made by the ERK and JNK signaling pathways, which can be activated in response to the binding of integrins to substrates [36].

EGFR can be stimulated by the binding of extracellular ligand or, in the absence of an extracellular ligand, via integrin signaling. In the case of integrin-dependent EGFR-phosphorylation, activation is dependent on the c-Src associated with the multimeric integrin complex [12]. c-Src-mediated phosphorylation of EGFR Tyr845 is involved in regulation of receptor function [13]. Adhesion-dependent phosphorylation sites on EGFR differ from growth factor-dependent phosphorylation sites [37]. Five autophosphorylation sites have been identified in the EGFR; all of them are clustered at the extreme carboxyl-terminal amino acids. Among these sites, Tyr^1068^, Tyr^1148^, and Tyr^1173^ are major sites [37]. We showed that EGFR is first phosphorylated at Tyr845 upon contact with bacteria. This indicates the direct involvement of c-Src in the activation of the EGFR. Indeed, the inhibition of c-Src reduced the intensity of *S. proteamaculans* invasion by 4 times.

### 3.2. c-Src, FAK and ILK Kinases in Bacterial Invasion

c-Src is recruited to the host-cell surface in response to the activation and autophosphorylation of FAK Tyr397, which leads to the formation of an active FAK-Src signaling complex [38]. c-Src kinase and FAK, in particular the FAK autophosphorylation site, are involved in promoting *Yersinia pseudotuberculosis* uptake [39]. FAK mediates the connection of ligand-bound integrin to dynamic actin–myosin networks and/or microtubule networks required for cell outgrowth [19]. The importance of FAK activation has been reported for pathogens that use integrins for invasion, including *Yersinia pseudotuberculosis* [39] and *Helicobacter pylori* [40]. Therefore, FAK is a very common target for many bacterial pathogens [40]. We have shown that FAK inhibition also reduces the intensity of *S. proteamaculans* invasion by 4 times.

Tyrosine kinases c-Src and FAK, when integrins and paxillin approach each other, participate in signal transduction through the phosphorylation of paxillin, creating additional docking sites for cytoskeleton assembly [41]. The approach of the cytoplasmic tail of β-integrins to paxillin, which has many docking sites for signaling molecules and actin-binding proteins, occurs as a result of the binding of both proteins to ILK [41]. ILK is a 59 kDa protein with a C-terminus that has homology with Ser/Thr kinases [42]. ILK plays key roles in connecting integrins to actin stress fibers, and transmitting various cellular signals through receptor tyrosine kinases to downstream integrins in response to extracellular matrix assembly [42]. In addition, ILK is required for the localization of IQGAP1 to the cell membrane, which then, together with mDia1, regulates local microtubule stability [43]. Integrin-ILK adhesions may bind microtubule plus ends to organize and stabilize them [44]. Microtubule rearrangements are necessary for *S. proteamaculans* invasion [45]. Impaired ILK kinase activity results in the significant reduction in the integrin-dependent invasion by *Streptococcus pyogenes, Staphylococcus aureus*, and *Yersinia* spp. [46]. We have shown that ILK inhibition reduces the intensity of *S. proteamaculans* invasion by 6–8 times.

### 3.3. α5β1 Intergin in Bacterial Invasion

Invading bacteria recruit c-Src kinase, which increases the efficiency of uptake, and FAK, which is necessary for uptake, to the site of contact as a result of integrin clustering [47]. We showed that α5β1 integrin is involved in *S. proteamaculans* invasion. Ingestion of bacterial pathogens by epithelial cells is also known to be initiated by either direct or indirect engagement with integrin receptors [46]. The natural ligand of α5β1 integrin is fibronectin. A variety of bacterial pathogens are known to interact with fibronectin, which in turn binds to integrins [6], including the human pathogens *Streptococcus pyogenes* [46] and *Campylobacter jejuni* [18]. However, fibronectin is not involved in *S. proteamaculans* invasion [7]. Bacterial proteins, such as Ipa of *Shigella flexneri* [48] and invasion of *Yersinia enterocolitica* and *Y. pseudotuberculosis* [49], are capable of directly interacting with α5β1 integrin. Thus, *S. proteamaculans* surface proteins may bind to α5β1 integrins without fibronectin, but the site of interaction is unknown.

### 3.4. Regulation of Invasion by RGD Peptide

The α5β1 integrin can be in multiple states, characterized by different strengths of binding to fibronectin. At the first stage of adhesion, it is in an intermediate state and provides a mechanical connection with fibronectin. The integrin is then activated, which leads to a significant increase in the adhesion strength. Increased binding strength occurs as a result of changes in integrin conformation and reflects distinct interactions between α5β1 integrin and sites on fibronectin [50]. In addition to differences in the interaction with ligand, these multiple conformational states may result in variations in intracellular regions of the integrin leading to different interactions with signaling and structural cytoskeletal components [50]. The extracellular heteromeric “head”, consisting of the N-terminal tails of the α and β integrin subunits, forms a pocket containing various recognition sites for short specific amino acid sequences of the ligand. The most well studied is the Arg-Gly-Asp (RGD) sequence, which is present in fibronectin, fibrinogen, vitronectin, and other extracellular matrix proteins [51]. In addition, it has recently been described that by triggering the release of ATP from eukaryotic cells, hemolysin ShlA of *S. marcescens* activates the P2Y2 receptor, which has an RGD domain that transactivates α5β1 integrins [52]. Addition of soluble RGD may result in the localization of α5β1 to focal adhesion complexes, likely causing changes in integrin conformation that allow interaction with cytoskeletal components [53]. And cells upon addition of RGD-coated beads form focal contacts enriched in activated c-Src and FAK kinases [54]. We hypothesized that the binding of RGD to integrins may trigger signaling mechanisms that increase the expression of receptors involved in bacterial invasion. But according to the RT-PCR data, the addition of RGD reduces the expression of α5, β1 integrins, and EGFR. The RGD domain interacts with both α5 and β1 subunits leading to changes in integrin conformation. This conformational change entails changes in the ligand binding interface, which determines the receptor-ligand binding [27]. Apparently, the RGD peptide changes the structure of the integrin such that after binding to RGD, the integrin interacts more closely with bacterial ligands.

### 3.5. Mechanism of S. proteamaculans Invasion

**Figure 6:**
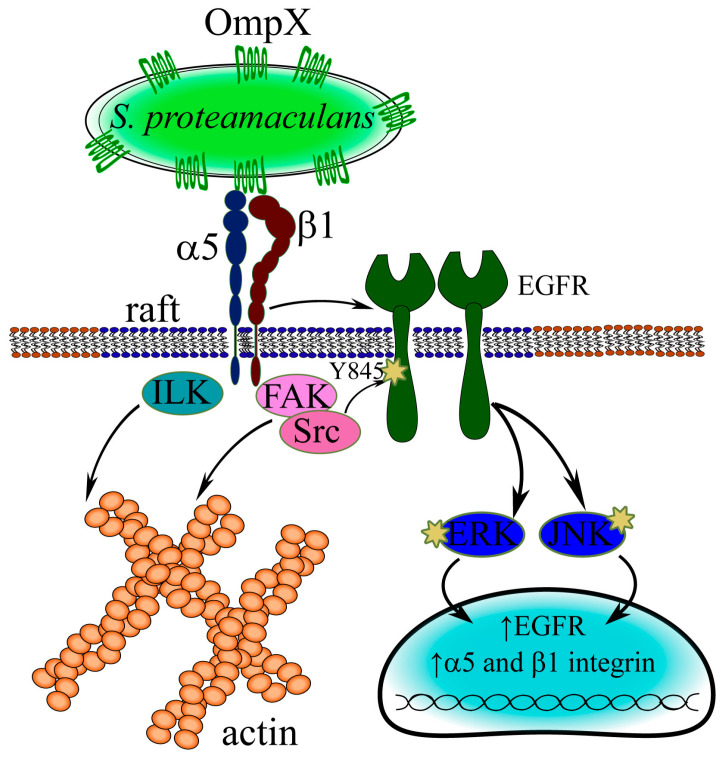
Schemes of interaction between *S. proteamaculans* and the host cell. The OmpX surface protein of *S. proteamaculans* binds to α5β1 integrin, which is anchored in a lipid raft with EGFR. Integrin binding activates FAK and ILK kinases, which mediate actin and tubulin rearrangements, and Src kinase, which phosphorylates EGFR at Tyr845. This leads to autophosphorylation of EGFR at Tyr1086. Phosphorylation is a signal for the transport of EGFR to the cell nucleus and the initiation of ERK and JNK signaling cascades. In this way, bacteria induce an increase in the expression of α5, β1 integrin, and EGFR involved in *S. proteamaculans* invasion.

Thus, using siRNA, we have shown that β1 and α5 integrins are involved in *S. proteamaculans* invasion. We have previously shown that bacteria colocalize with these receptors during infection [21]. For each specific integrin, there are at least three types of conformation corresponding to different activation states: “bent closed”, “extended closed”, and “extended open”. RGD binding promotes the conformational activation of the receptor from bent to extended states, facilitating receptor binding to a ligand lacking the RGD domain [27]. In this work, we have shown that in the presence of the RGD peptide, *S. proteamaculans* adhesion increases by 30%, indicating the direct binding of bacterial proteins to integrins. In addition, the RGD peptide additionally enhances the expression of the β1 and α5 subunits of host-cell integrins in response to *S. proteamaculans* infection. Increased expression of the β1 receptor occurs in response to contact with the surface protein OmpX of *S. proteamaculans* [7]. Thus, OmpX interacts with integrins (Figure 6). And the participation of α5 and β1 integrin subunits in the *S. proteamaculans* invasion allows us to assume that the receptor of OmpX is α5β1 integrin. We have shown that FAK and ILK kinases, which transmit signals from integrins and ensure actin and tubulin rearrangements, are involved in the invasion of *S. proteamaculans*. In addition, invasion is reduced by an inhibitor of Src kinase, which is a component of the integrin–EGFR complex, and by the inhibitor AG1478, which leads to the breakdown of this complex [55]. Upon infection with bacteria, EGFR is first phosphorylated at Tyr845, which is phosphorylated by Src kinase, and only then at Tyr1086, which can be phosphorylated in response to the ligand binding of both EGFR itself and integrins that form a complex with EGFR [55]. In addition, EGFR is transported from the membrane to the cell nucleus, but colocalization of bacteria with EGFR-containing endosomes was not detected. This also indicates that the EGFR receptor is involved in signal transduction rather than interacting directly with bacterial surface proteins. Then, using ERK and JNK signaling cascades, the bacteria induce a host-cell response that promotes invasion.

## 4. Materials and Methods

### 4.1. Cell Cultures, Bacterial Strains, and Growth Conditions

The cervical carcinoma M-HeLa and adenocarcinoma alveolar basal epithelial A549 cell lines were obtained from the “Vertebrate cell culture collection” (Institute of Cytology, St. Petersburg, Russia). M-HeLa cells were grown in MEM medium contained 1% nonessential amino acids (NEAAs) (Sigma-Aldrich, Munich, Germany) and 10% fetal bovine serum (Sigma-Aldrich, Munich, Germany). A549 cells were grown in DMEM medium contained 10% fetal bovine serum (Sigma-Aldrich, Munich, Germany).

*S. proteamaculans* strain 94 was isolated as described earlier [56] and grown as previously described [2].

### 4.2. siRNA Transfection

The expression of host-cell proteins was inhibited using siRNA targeting α5 integrin (sc-29372) and β1 integrin (sc-35674) (Santa Cruz Biotechnology, Dallas, TX, USA). Transfection of siRNAs was performed using siRNA Transfection Reagent (sc-29528) as recommended by the manufacturer (Santa Cruz Biotechnology, Dallas, TX, USA). The RNA interference efficiency was controlled by Western blotting as previously described [7].

### 4.3. Western Blot Analysis

After transfection of siRNAs or incubation with *S. proteamaculans*, cells were incubated with electrophoresis sample buffer (4% SDS, 24% glycerol, 200 mM DTT, 0.01% bromophenol blue, 125 mM Tris-HCl, pH 6.8) for 5 min at 56 °C. Cells were scraped off the plate, followed by a 5 min boiling in the TDB-120 thermostat (Biosan, Rīga, Latvia). The samples were fractionated by SDS-PAGE and transferred to a Hybond ECL membrane according to the manufacturer’s instructions (GE Healthcare, Chalfont Saint Giles, UK). The membrane was incubated with 5% nonfat milk in PBS 40 min to prevent nonspecific binding of antibodies and then incubated with rabbit primary antibodies against EGFR [E235] at a dilution of 1:1000 (Abcam, Cambridge, UK), β1 integrin [EPR16895] at a dilution 1:1000 (Abcam, Cambridge, UK), α5 integrin [EPR7854] at a dilution of 1:500 (Abcam, Cambridge, UK), and GADPH at a dilution 1:1000 (Abcam, Cambridge, UK) at room temperature for 2 h. The membrane was then washed three times with washing buffer (5% nonfat milk, 0.1% Tween 20, PBS) for 10 min, incubated for 1 h with the secondary antibodies (1:20,000) against rabbit IgG (Thermo Fisher Scientific, USA) and mouse IgG (Abcam, Waltham, MA, USA) conjugated with horseradish peroxidase. The blots were washed with washing buffer three times and developed using SuperSignal West FEMTO Chemiluminescent Substrate (Thermo Fisher Scientific, Waltham, MA, USA) according to the manufacturer’s recommendations.

### 4.4. Quantitative Invasion Assay

Efficiency of invasion was evaluated by the quantitative invasion assay [57] with some modifications. Cells forming a 50–70% monolayer were transfected with RNA or treated with inhibitors in DMEM before adding bacteria. To inhibit the EGFR, the cells were pretreated with 30 μM tyrphostin AG-1478 (Sigma-Aldrich, Munich, Germany) for 1 h before adding bacteria. To inhibit the c-Src kinase, the cells were pretreated with 5μM Src I1 (Sigma-Aldrich, Munich, Germany) for 1 h before adding bacteria. To inhibit the FAK, the cells were pretreated with 5 μM Y15 (Sigma-Aldrich, Munich, Germany) for 1 h before adding bacteria. To inhibit the ILK, the cells were pretreated with 2.5 μM cpd22 (Sigma-Aldrich, Munich, Germany) for 24 h before adding bacteria. To inhibit the Raf/MEK/ERK signaling pathway, the cells were pretreated with 10 μM U0126 (selective inhibitor of MAP kinase kinases, MEK1 and MEK2) (Sigma-Aldrich, Munich, Germany) for 1 h before adding bacteria. To inhibit the JNK signaling pathway, the cells were pretreated with 10 μM SP600125 (Sigma-Aldrich, Munich, Germany) for 1 h before adding bacteria. Bacteria were grown for 48 h until the actinase activity in the extracts of the wild-type *S. proteamaculans* could be determined [2]. The total amount of bacteria was determined from the suspension optical density at 600 nm. Bacteria in serum-free DMEM were added to the eukaryotic cells at a ratio of approximately 10,000 bacteria per cell [16]. After co-cultivating host cells and bacteria, the proportions of viable cells were measured after staining with trypan blue by counting in a Goryaev chamber. Cell viability in all experiments was at least 90%. After co-cultivating host cells and bacteria at 37 °C in 5% CO_2_ for 2 h, suspension of the infected cells was incubated in DMEM containing kanamycin (100 μg/mL) with shaking at 37 °C for 1 h, to kill extracellular bacteria. Then, the cells were lysed with 1.5% sodium deoxycholate, quickly diluted with cold LB medium 100 times and the aliquots of the resulting suspension (100 μL) were plated on LB-agar to determine the number of colony forming units (CFU) corresponding to the of intracellular bacteria. The intensity of invasion was determined as the number of CFU normalized to the number of viable eukaryotic cells determined after incubation with bacteria. The results for each experiment were the average of an assay performed in triplicate and independently repeated three times.

### 4.5. Fluorescence Microscopy

Cells were grown at 37 °C in an atmosphere of 5% CO_2_ on coverslips until a 70–80% monolayer was formed. If necessary, before the experiment, the cells were incubated with 5 μg/mL or 50 μg/mL RGD peptide for 30 min. The ACDVRGDCFCG (RGD) peptide was obtained and purified by Alex Komelev (Institute of Experimental Medicine, St. Petersburg, Russia). *S. proteamaculans* were grown in LB medium (Sigma-Aldrich, Munich, Germany) with aeration for 46 h at 30 °C. The bacterial suspension was centrifuged at 9600 g, 8 min. The pellet was resuspended in DMEM medium and added to eukaryotic cells at a ratio of 10,000 bacteria per cell. Bacteria were deposited on the surface of the host cell by centrifugation for 5 min at 560× *g*. The host cells and bacteria were co-cultivated at 37 °C in 5% CO_2_ for 2 h. Samples were stained and analyzed as previously described [7]. Eukaryotic cell receptors were stained with the rabbit primary antibodies against α5 integrin [EPR7854] at a dilution of 1:500 (Abcam, Cambridge, UK) and the mouse antibodies against β1 integrin [P5D2] at a dilution of 1:250 (Abcam, Cambridge, UK). Antibodies against EGFR phosphorylated at Tyr845 [EPR2149Y] and phosphorylated at Tyr1086 [Y39] at a dilution of 1:200 and 1:100 (Abcam, Cambridge, UK), respectively, were used to stain phosphorylated EGFR. After washing with 0.05% Tween-ZBS solution, goat anti-mouse IgG-FITC secondary antibodies (#sc-2010, Santa Cruz Biotechnology, Dallas, TX, USA) at a dilution of 1:500 were added to the preparations stained for β1 integrin, and goat anti-rabbit IgG-FITC secondary antibodies (#sc-2012, Santa Cruz Biotechnology, USA) at a dilution of 1:1000 were added to all other preparations. Cells stained with anti-species antibodies only were used as a negative control. The preparations were analyzed using an Olympus FV3000 microscope (Tokyo, Japan) using a system of lasers with wavelengths of 405 (blue fluorescence), 488 (green fluorescence), and 561 nm (red fluorescence).

### 4.6. Real-Time RT-PCR

Total RNA was extracted from cells using Dia-M Extraction Kit according to the manufacturer’s instructions (Dia-M, Moscow, Russia). Gene expression was assessed using CFX96 Touch Real-Time PCR Detection Systems (Bio-Rad, Hercules, CA, USA) as described previously [16]. Target gene expression was normalized to the expression of a cellular housekeeping gene, β-actin or GADPH, and calculated using the 2^−ΔΔCT^ method. Gene-specific primer pairs (Evrogen, Moscow, Russia) designed using BLAST-primer software (https://www.ncbi.nlm.nih.gov/tools/primer-blast/, accessed on 17 August 2018) and used for real-time PCR are listed in Table 1. Qualitative analysis prior to the Real-Time PCR confirmed that only one fragment with a molecular weight calculated using BLAST software was synthesized from the cDNA template with gene-specific primer pairs.

### 4.7. Statistical Analysis

Each quantitative experiment was repeated 3–5 times with three repetitions in each experiment. In the figures, values are expressed as the mean ± S.D. (error bars) of a representative experiment. Significance testing in comparisons is based on Student’s *t*-tests for pairs and analysis of variance (ANOVA) with Excel Data Analysis Pack. The difference was considered significant at the *p* < 0.05 level.

## Figures and Tables

**Table 1:** Gene-specific primer pairs.

Target Gene	Primer Sequences
*α5 integrin*	Forward 5′-GGCTTCAACTTAGACGCGGA-3′
	Reverse 5′-AAGCCTCCTTGGCAGTAACC-3′
*β1 integrin*	Forward 5′-GACGCCGCGCGGAAAAG-3′
	Reverse 5′-ATCTGGAGGGCAACCCTTCT-3′
*EGFR*	Forward 5′-GTGCAGCTTCAGGACCACAA-3′
	Reverse 5′-AAATGCATGTGTCGAATATCTTGAG-3′
*β-actin*	Forward 5′-AATCTGGCACCACACCTTCTACA-3′
	Reverse 5′-GACGTAGCACAGCTTCTCGTTA-3′
*GADPH*	Forward 5′-GGCATGGACTGTGGTCATGAG-3′
	Reverse 5′-TGCACCACCAACTGCTTAGC-3′

## Data Availability

The original contributions presented in this study are included in the article. Further inquiries can be directed to the corresponding author.
